# Porcine Ovarian piRNA Dynamics: A Comparative Study During Follicular Atresia

**DOI:** 10.3390/biology14060609

**Published:** 2025-05-26

**Authors:** Jinbi Zhang, Long Huang, Xinxin Qin, Wenjie Li, Xiaolong Cheng, Zengxiang Pan

**Affiliations:** 1 College of Animal Science and Food Engineering, Jinling Institute of Technology, Nanjing 211169, China; zhangjinbi@jit.edu.cn; 2 College of Animal Science and Technology, Nanjing Agriculture University, Nanjing 210095, China; njauhl@163.com (L.H.); 2022105033@stu.njau.edu.cn (X.Q.); 2023105025@stu.njau.edu.cn (W.L.); 2023805139@stu.njau.edu.cn (X.C.)

**Keywords:** piRNA, follicle atresia, granulosa cells, high-throughput sequencing

## Abstract

Follicular atresia is a natural process of follicular degeneration in mammal ovaries, significantly impacting female reproductive potential. However, the underlying regulatory mechanisms remain underexplored, particularly those involving non-coding RNAs like PIWI-interacting RNAs (piRNAs). In this study, we collected single antral follicles from the ovaries of 180-day-old commercial sows, classified them as healthy (HF) and atretic (AF) based on morphological and biochemical criteria, and sequenced the RNA samples using the Illumina Hiseq 3000 system (San Diego, CA, USA). piRNAs were identified using three algorithms, and the differential expression was compared and validated by qPCR. The target genes of differentially expressed piRNAs were predicted and subjected to functional analysis. A total of 452 piRNAs were identified across all samples, with 103 showing differential expression between HFs and AFs. Among the top 12 piRNAs with the most significant expression differences validated by qPCR, 5 (piR-23, piR-27, piR-64, piR-65, and piR-76) exhibited statistically significant differences. Pathway analysis showed that these piRNAs primarily targeted genes involved in cell apoptosis regulation, inflammation and oxidative stress response, substance transport and signal transduction, and cellular structural integrity maintenance. Our study provides the first comprehensive profile of piRNAs in porcine ovarian follicles during atresia and reveals underlying potential regulatory mechanisms. These findings enhance our understanding of piRNA functions during the early follicular atresia process and offer insights for further functional studies and biomarker development in ovarian pathology.

## Simple Summary

This study explores the role of piRNAs in porcine ovarian follicles during atresia. We used high-throughput sequencing to identify piRNAs in healthy and early atretic antral follicles and further analyzed the target genes that interact with them and their related pathways. The results show that piRNAs might be involved in various processes, including cell apoptosis, inflammation, oxidative stress, substance transport, signal transduction, and cell structure maintenance. This research provides new insights into the ncRNA-involved regulatory mechanisms in ovarian cells, advances our understanding of piRNA functions during early follicular atresia, and could be helpful in improving pig breeding and ovarian health issues.

## 1. Introduction

The ovarian follicle reserve in mammals, including pigs, is established during the fetal period and represents the female’s total reproductive potential. Throughout all the stages of follicular development, a physiological process of ovarian follicle degeneration and resorption occurs, which is termed follicular atresia. In this process, a large number of follicles degenerate and do not proceed to ovulation. Follicular atresia is a normal physiological process in female animals, while abnormal physiological and pathological conditions can lead to the excessive occurrence of follicular atresia [1].

In pigs, the first significant atresia peak is observed in follicles that have reached approximately 1 mm in diameter during the antral stage. The atresia rate further escalates when the rest of the follicles grow to 3–5 mm in diameter [2], ultimately limiting the number of ovulated oocytes [3]. A pivotal factor contributing to follicular atresia is the apoptosis of granulosa cells (GCs), which are the primary cells that nourish the developing oocyte and are essential for follicle growth. The balance between the survival and death of GCs is intricately regulated by a complex array of growth factors and pro-apoptotic signals [4]. This molecular regulatory process encompasses both the transcriptional activation of genes and post-transcriptional mechanisms that influence mRNA stability and protein synthesis [5,6]. Unraveling the sophisticated regulatory networks that govern GCs apoptosis and follicular atresia is a significant pursuit in reproductive biology.

Over the past few decades, the rapid advancement of research on non-coding RNAs (ncRNAs) has revolutionized reproductive system studies by uncovering new regulatory layers. In domestic animals, the ncRNA study has garnered considerable attention. In pigs, genome-wide changes in microRNAs (miRNAs) and circular RNAs (circRNAs) associated with the atresia process were identified in medium-sized antral follicles [6,7,8], and specific regulatory networks of particular miRNA and circRNAs were revealed through post-transcriptional gene silencing [9,10]. Interestingly, another class of less studied ncRNAs has come to our attention. In 2006, scientists discovered that certain gene mutations in *Drosophila melanogaster* caused male flies to exhibit phenotypes such as reduced testis size and infertility. These genes were named the PIWI (P-element-induced wimpy testis) family of genes. Subsequently, RNAs that interact with PIWI proteins were named PIWI-interacting RNAs (piRNAs) [11]. piRNAs are a class of recently discovered small ncRNAs with a typical length between 24–32 nt which play a role in forming RNA-induced silencing complexes (RISCs) with PIWI-clade argonaute proteins. Two biogenesis pathways of piRNAs were later identified. Primary piRNAs are derived from long precursor transcripts that are processed and loaded onto PIWI proteins. In contrast, secondary piRNAs are produced through a “ping-pong” amplification loop, where Piwi proteins with primary piRNAs target complementary transcripts, generating secondary piRNAs with a 10 nt offset [12]. Generally, the regulatory pathway of the piRNAs was considered a conserved defense mechanism that protects genetic information from harmful effects by targeting molecular parasites such as active transposons [13,14]. Recently, researchers have demonstrated that the piRNAs machinery also regulates protein-coding genes via miRNA-like mechanisms, which degrade mRNA transcripts through direct binding [15]. These findings greatly expanded the possible function of piRNAs.

In pigs, the profiling and comparative analysis of piRNAs have been conducted across various stages of sexual maturity, particularly in the testes. These studies have demonstrated the pivotal role of piRNAs in the regulation of porcine spermatogenesis [16,17]. In addition, comparing piRNA expression between adult porcine ovaries and testes revealed a small (2.6%) overlap of piRNAs between the female and male gonads [18]. However, there has been no detailed description of piRNAs in the porcine follicles, especially during the typical atresia process. In this study, we explored the expression patterns of piRNAs between healthy (HF) and atretic (AF) porcine ovarian follicles using Illumina sequencing analysis. This research provides new insights into the expression patterns and characteristics of piRNAs during the early atresia of porcine ovarian follicles, provides candidate piRNAs for related functional studies and biomarker evaluations, and offers insights for studies of ovarian pathology.

## 2. Materials and Methods

### 2.1. Follicle Collection and Classification

The ovaries were obtained from healthy 180-day-old commercial sows (Duroc × Yorkshire × Landrace) at a local slaughterhouse in Huai’an, Jiangsu. The ovaries were washed twice with 75% ethanol and physiologic saline and then transported to the laboratory in PBS at 4 °C.

Individual follicles measuring 3 to 5 mm in diameter were dissected from the ovaries using small scissors and fine forceps under a surgical dissecting microscope (SZ51; Olympus, Tokyo, Japan) and were then classified as healthy follicles (HFs) and atretic follicles (AFs), according to criteria described in our previous studies, which combined morphological and biochemical standards [5]. Briefly, ovarian follicles with a clear and transparent appearance, visible small blood vessels, low GC density (≤250 cells/μL), and a low P4/E2 ratio (≤5) were categorized as HFs. Ovarian follicles with a turbid orange appearance, visible large blood vessels, high GC density (250–1000 cells/μL), and a high P4/E2 ratio (5–10) were categorized as AFs. For the Illumina deep sequencing, we selected 9 HFs and 9 AFs (without specifically distinguishing whether they originated from different animals). We pooled three follicles to form a mixed sample, resulting in three biological replicates for both the healthy follicle group (designated as H1, H2, and H3) and the early atretic follicle group (designated as EA1, EA2, and EA3), which ensures the reliability and reproducibility of our sequencing results. Additionally, GCs from 20 individual follicles, comprising 10 HFs and 10 AFs, were selected for qRT-PCR validation. The GCs sample was prepared by pooling two cell populations: (1) residual lumenal GCs post-cell counting and (2) mural GCs mechanically detached from the follicular wall with forceps.

### 2.2. Small RNA Library Preparation and Illumina Sequencing

Total RNA from the follicle samples was extracted using Trizol reagent (Invitrogen, Carlsbad, CA, USA), following the manufacturer’s instructions. Total RNA quantity and purity were determined using a Bioanalyzer 2100 (Agilent, Palo Alto, CA, USA); all samples exhibited an RIN > 7.0. Small RNA libraries were constructed following the protocols of the TruSeq Small RNA Sample Prep Kit (Illumina, San Diego, CA, USA). Single-end sequencing (50 bp) was performed on an Illumina Hiseq 3000 system.

### 2.3. Identification of piRNAs

After the preliminary filtration processes, the obtained clean reads were aligned to the pig reference genome (Sus scrofa 11.1) using the BWA tool [19,20]. Three algorithms, Piano [21], piRNApredictor [22], and proTRAC [23], were applied to screen and identify the expression of piRNAs in HFs and AFs. Both Piano and piRNApredictor exhibit high accuracy and sensitivity, while proTRAC is primarily used to detect piRNA clusters. The intersection of these three identification tools allows for the more accurate identification of the required piRNAs.

### 2.4. Differential Expression of piRNAs

The expression level of piRNAs in this study is calculated using the RPM (the number of reads per million clean tags) method, which calculates the number of reads that map to the pig genome per million clean reads. We measured the differential expression of the piRNAs between HFs and AFs with DESeq2 in R v4.1.0 [24]. We considered the piRNAs to be differentially expressed if the absolute value of log2 fold change was greater than 1 (i.e., at least a 2-fold difference) and the adjusted *p*-value (Benjamini and Hochberg’s method) was <0.05.

### 2.5. Biological Functions Analysis of Potential Target Genes

To identify mRNAs that may be degraded through piRNA-mediated cleavage pathways, we mapped the obtained piRNAs to mRNA sequences of the pig species using the short sequence alignment tool Bowtie [24], using specific methods suggested in the research work of Peng et al. [25]. Then, ClueGO was applied to analyze the obtained target genes and perform a biological function analysis of the target genes related to differentially expressed piRNAs, mainly including GO analysis and pathway enrichment analysis of regulatory pathways.

### 2.6. qPCR Analysis

To confirm the expression levels of piRNAs in the follicles, we applied a microRNA reverse transcription kit (EZBioscience, Roseville, MN, USA) to reverse-transcribe total RNA into cDNA, following the manufacturer’s instructions. qPCR was performed using SYBR Premix Ex Taq (Takara, Dalian, China) to detect the expression of piRNAs. Primers were designed according to the method for microRNA tailing primers [24]. U6 small nuclear RNA was used as an endogenous reference gene for normalization. The amplification primer sequences for each piRNA and U6 are listed in Table S1. The thermal cycling conditions were as follows: initial denaturation at 95 °C for 10 s, followed by 40 cycles of denaturation at 95 °C for 10 s, and annealing and extension at 58 °C for 60 s. Melting curve analysis was performed from 60 °C to 95 °C to verify the specificity of the amplified products. The 2^−ΔΔCt^ method was used to analyze the relative expression levels of piRNAs. The qPCR data were analyzed using GraphPad Prism 9 (GraphPad Software, San Diego, CA, USA). Statistical differences were determined using an unpaired *t*-test, with values of *p* < 0.05 considered statistically significant. Each experimental point on the graph represents the mean ± SE of at least three technical replicates.

## 3. Results

### 3.1. Sequencing Data Overview and Quality Control Analysis

**Figure 1:**
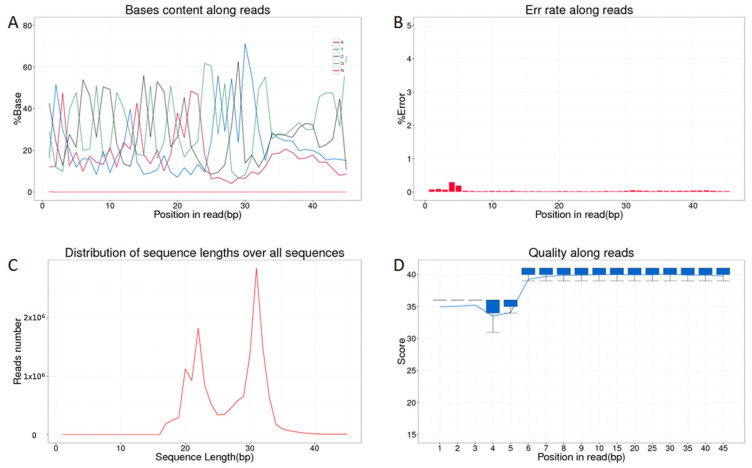
The quality control analysis of the clean data (represented by HF1). (**A**) Clean reads base composition distribution; (**B**) clean reads base error rate distribution; (**C**) clean reads base length distribution; (**D**) clean reads base quality distribution.

Overall, a total of 104,341,090 raw sequence reads were obtained from sequencing, including an average of 16,714,427 from HFs and 18,065,936 from AFs, respectively. After removing the adapters and discarding sequences shorter than 17 nt or longer than 35 nt, 95,238,765 clean reads (91.28%) remained for further analysis. The detailed sequencing data statistics are shown in Table S2. The clean reads were then subjected to alignment against the porcine reference genome, and it was found that all the alignment rates exceeded 97%, indicating a high degree of sequence concordance (Table 1). The quality control analysis of the sequencing data is shown in Figure 1. According to the results, most reads cluster around the typical length of piRNA, which is 24–32 bp. The base content shows no significant sequencing bias, while the error rate distribution and quality scores are consistent and indicate good data quality.

### 3.2. Screening of Porcine Ovarian piRNAs

**Figure 2:**
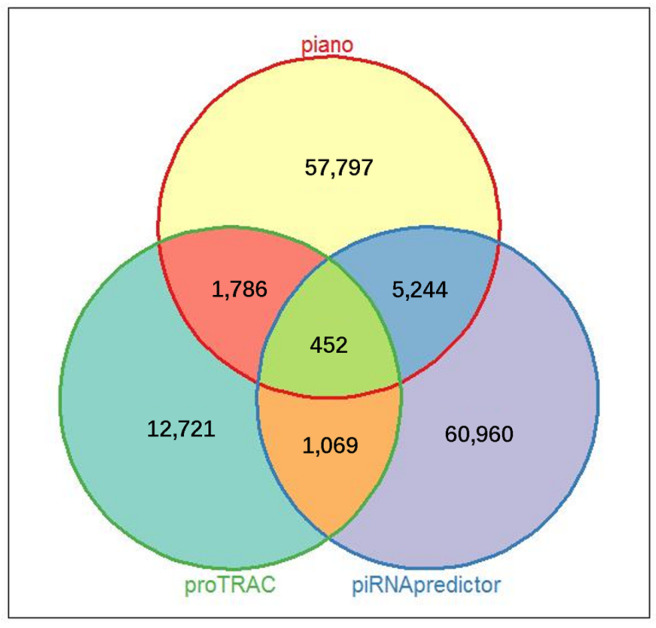
The intersection of piRNA detection across all the samples using the three algorithms.

A comparative analysis was conducted to identify porcine ovarian piRNAs using three distinct bioinformatics algorithms: Piano, piRNApredictor, and proTRAC. The outcomes of this analysis revealed a significant disparity in the number of piRNA molecules identified by each algorithm. Specifically, Piano and piRNApredictor reported 65,278 and 67,725 piRNAs, respectively, substantially higher than the 16,027 piRNAs detected by the proTRAC method (as detailed in Table S3 and Figure S1). To ensure the accuracy of the screening results, we selected the intersection of the outcomes from the three algorithms (Figure 2), and a number of 452 piRNAs were identified across all the porcine follicular samples.

### 3.3. Differential Expression Profile of piRNAs in Porcine HFs and AFs

**Figure 3:**
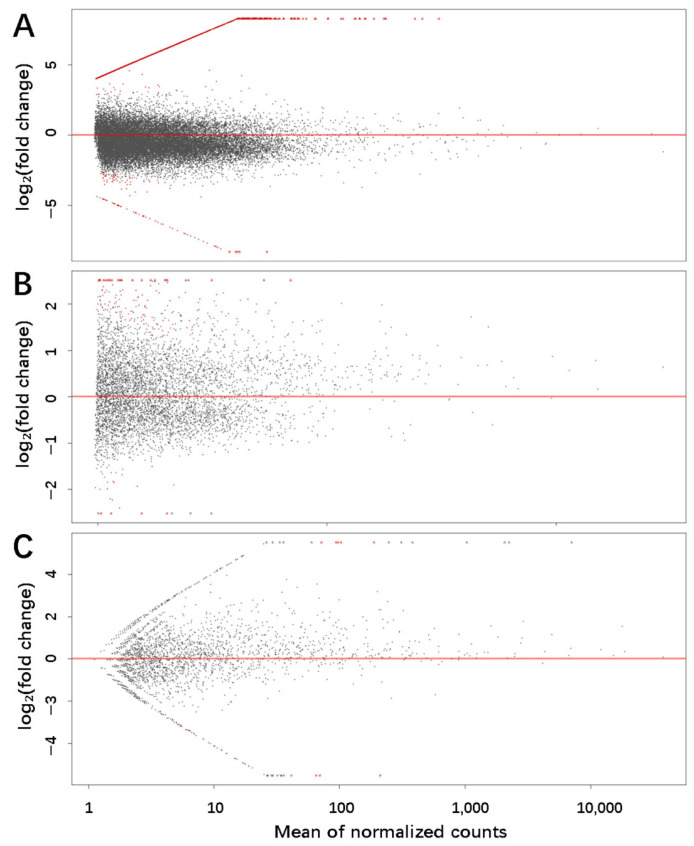
Differential expression profiles of piRNA molecules. (**A**) differentially expressed piRNAs generated by Piano; (**B**) differentially expressed piRNAs generated by piRNA predictor; (**C**) differentially expressed piRNAs generated by proTRAC. Note: Red points represent genes that are significantly differentially expressed, black points indicate genes that do not meet the criteria for significant differential expression.

To compare the expression of piRNAs between HFs and AFs, the piRNAs with an absolute value of log2 fold change greater than 1 (greater than 2-fold difference) and the adjusted *p*-value < 0.05 were considered to be differentially expressed. We identified 2414, 633, and 296 differentially expressed piRNAs with the piRNA lists generated by Piano, piRNA predictor, and proTRAC, respectively (Figure 3). A total of 103 piRNAs were found to be differentially expressed and met the criteria of at least two of the algorithms.

### 3.4. qPCR Confirmation of Differentially Expressed piRNAs

**Figure 4:**
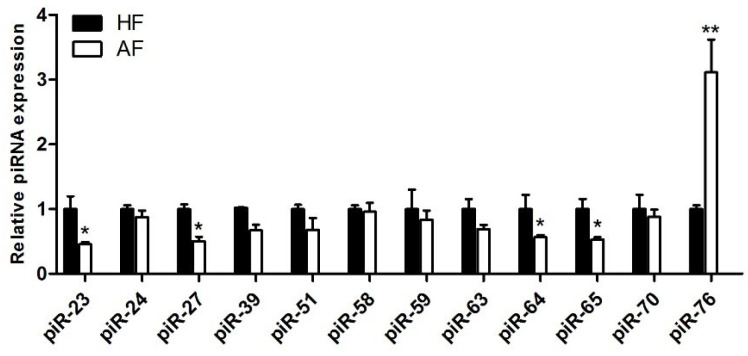
Expression of piRNA in porcine healthy follicles and early atretic follicles. Each experiment comprises three independent repetitions, and results are shown as mean ± SEM. * indicates a significant difference, *p* < 0.05. ** indicates a very significant difference, *p* < 0.01.

Among the differentially expressed piRNAs, we selected 12 specific piRNAs (Table S4) identified as piRNAs in all three algorithms, showing the greatest expression differences between HFs and AFs. These are predominantly concentrated between 26–28 nt, and 11 showed higher expression levels in HAs.To verify their expression, we isolated 10 HFs and 10 early Afs, as described previously, and used qRT-PCR technology to validate the expression of these piRNAs. According to the results (Figure 4), the expression trends of all piRNAs were completely consistent with the high-throughput sequencing results. Five piRNAs, termed piR-23, piR-27, piR-64, piR-65, and piR-76, showed significant differences.

### 3.5. Prediction of piRNA Target Genes

**Figure 5:**
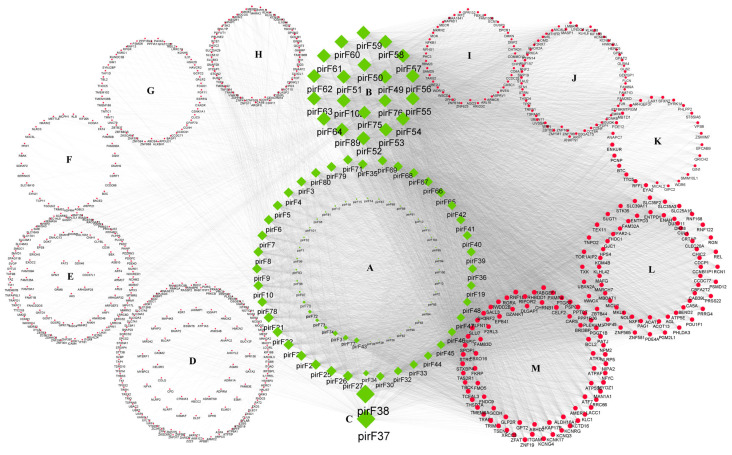
Prediction of piRNA target genes. The green nodes in the figure represent differentially expressed piRNA molecules (**A**–**C**), and the red nodes represent target genes (**D**–**M**). The size of the node represents the number of targeted genes. The larger the node, the higher the interaction frequency. Custom-define code: “pir” indicates piRNA, “F” indicates follicle, and the numbers indicate the identified piRNA ID numbers.

The previously screened 103 piRNAs (Table S5), which exhibited differential expression between HFs and AFs, were aligned to all pig mRNA sequences using Bowtie, and 638 related target genes were associated with piRNAs, as shown in Figure 5 and Table S6. It can be seen that piR-37 and piR-38 display a relatively high number of potential target genes, while genes such as AKAP17B (A-kinase anchoring protein), ALDH16A1 (aldehyde dehydrogenase), BCL2 (B-cell CLL/lymphoma 2), etc. are targeted by a relatively high number of piRNAs.

### 3.6. Functional Analysis of Target Genes

**Figure 6:**
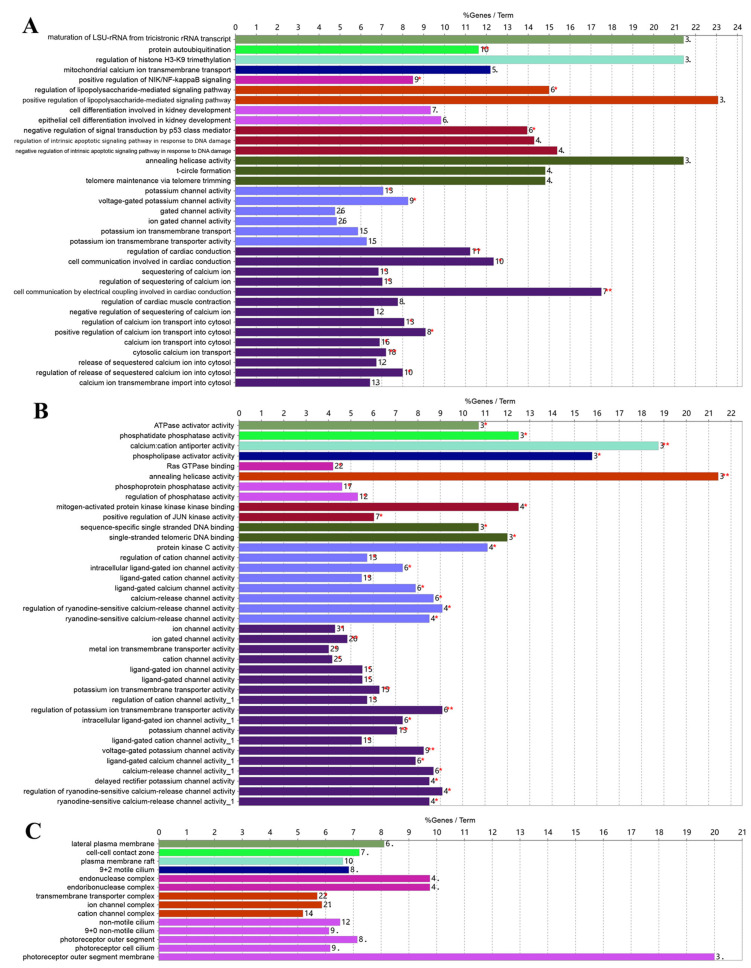
Gene ontology (GO) analysis of piRNA target genes. The biological process (**A**), molecular function (**B**), and cellular component (**C**) are generataed by gene ontology (GO) analysis. The vertical axis represents the GO term classification, the horizontal axis represents the percentage of the matched genes in the total number of genes of the GO term, the numbers at the end of the bars of different colors represent the number of matched genes, and the same color represents the related GO term. *: *p* < 0.05 and **: *p* < 0.01.

The biological function and pathway analysis was conducted on the target genes related to differentially expressed piRNAs. As presented in Figure 6, gene ontology (GO) analysis highlighted the transportation of potassium and calcium ions; epigenetic regulation, including histone H3K9 trimethylation and mitochondrial calcium ion transmembrane transport; as well as apoptosis regulation, such as negative regulation of signal transduction by p53and protein autoubiquitination, which hinted at a role of piRNAs in GCs apoptosis during atresia.

**Figure 7:**
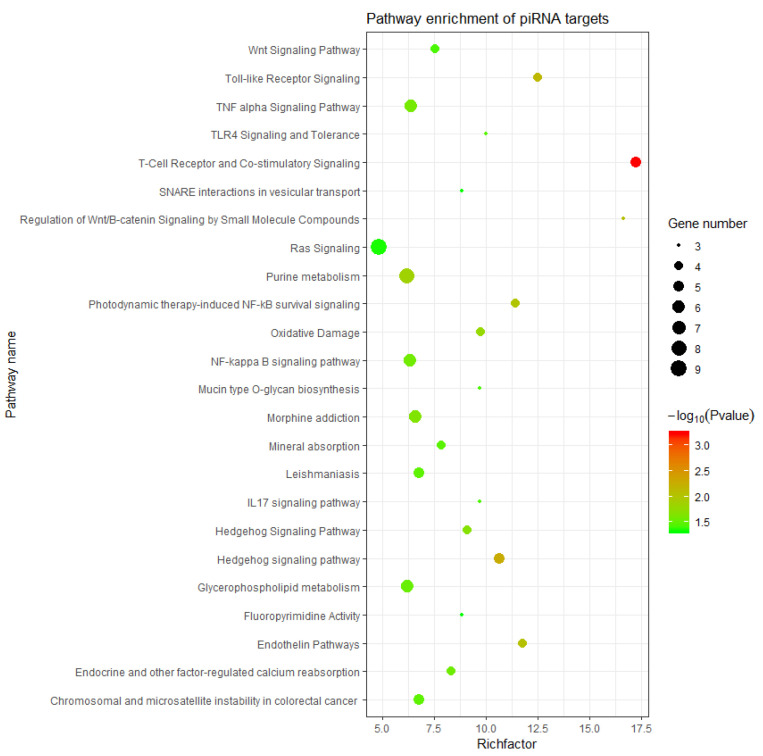
KEGG enrichment bubble chart. The size and color of each bubble in the figure represent the comparison of number of genes enriched to the pathway and the size of the enriched *p* value.

In addition, the KEGG pathway enrichment analysis was presented using a bubble chart, as shown in Figure 7. The result encompasses a broad range of biological pathways, suggesting piRNAs’ critical role in various aspects of cellular function and regulation, from signal transduction to metabolism. Most interestingly, the Wnt signaling pathway, hedgehog signaling pathway, and oxidative damage NF-kappa B signaling pathway were observed. These pathways hinted that piRNAs are involved in cell fate determination and cell growth and differentiation, as well as in the responses to oxidative stress and antioxidant defenses during follicular atresia.

## 4. Discussion

Since the discovery of piRNAs, they have attracted significant attention due to their functions in germline and reproductive physiology. In domestic animals, piRNAs have been characterized in sheep testes and ovaries [26], as well as in porcine oocytes and early embryos [27]. Here, for the first time, we profiled piRNAs in porcine follicles during atresia. In general, three algorithms generated 12721-60960 piRNAs, respectively. These piRNAs may bind to the mRNAs of target genes, promoting their degradation or inhibiting their translation, thereby regulating their expression [28]. In this way, during the process of follicular atresia, the piRNAs with differential expression may affect the expression levels of target genes and further influence the development and atresia process of follicles. Based on our findings, the differentially expressed piRNAs are likely to be involved in several potential processes discussed below.

### 4.1. The Regulation of Cell Apoptosis

It is well-accepted that the apoptosis of GCs is an essential cause of follicular atresia [29]. Although the small RNA library preparation was carried out in the whole follicle, qPCR in GCs confirmed that the expression trends of the corresponding genes in the GCs were consistent with those observed at the level of the whole follicle. This, to some extent, also implies the dominant role of GCs in follicular atresia. As revealed by KEGG enrichment results, Wnt pathway and oxidative damage have long been proven to be important molecular mechanisms in the regulation of GCs apoptosis, and their changes and functions in follicular atresia have been verified through previous studies [29,30,31].

Some other genes were also highlighted in our study. Specifically, the BCL2 (B-cell CLL/lymphoma 2) family comprises a large number of members. Several members of the BCL2 family, including both pro-apoptotic BIM (Bcl-2 interacting mediator of cell death) and anti-apoptotic BCL2, are on the list of target genes of the differential piRNAs. These proteins affect cell apoptosis by modulating the permeability of the mitochondrial membrane, the homeostasis of intracellular calcium ions, and oxidative stress [32]. In addition, RNF183 (RING finger protein 183) and TRIM13 (tripartite motif containing 13), which play a role in the ubiquitination process, were also noticed. Since the ubiquitination process is essential in the regulation of the stability of apoptosis-related proteins [33], we can reasonably speculate that piRNAs are involved in a precisely regulatory process of the apoptosis signaling pathway, thereupon determining follicular atresia by disrupting the balance between cell survival and apoptosis.

### 4.2. Inflammation and Oxidative Stress

There is a complex regulatory relationship between the inflammatory response and apoptosis. Additionally, oxidative stress can not only directly damage cells and trigger apoptosis, but it can also activate inflammatory signaling pathways. In this study, genes such as PTGS2 (prostaglandin-endoperoxide synthase 2), ALDH16A1 (aldehyde dehydrogenase 16A1), and SLFN11 (Schlafen 11) that appear in the target gene list imply an involvement of piRNAs in inflammation and oxidative stress. PTGS2 is a key enzyme for synthesizing inflammatory mediators such as prostaglandins. Its anti-apoptotic and pro-apoptotic effects have been extensively studied in cancer, as well as in the liver and vascular smooth muscle cells. A recent study suggests that it promotes estradiol synthesis and anti-apoptosis in porcine GCs [34], which confirms our assumption. There is not much research on ALDH16A1; however, as an aldehyde dehydrogenase, it belongs to a group of NAD(P)+-dependent enzymes involved in the biosynthesis of retinoic acid and a wide range of metabolic functions. Other members of this family play a clear role in resisting oxidative stress damage [35,36], which hints at the anti-apoptotic role of ALDH16A1. Lastly, SLFN11 has been proven to respond to DNA damage in studies of various cancer cells [37]. By binding to chromatin, SLFN11 causes a replication block and induces apoptosis [38]. Combined with the conclusion in the study of the human retina that piRNAs were dysregulated under oxidative stress conditions [39], we can reasonably speculate that piRNAs are involved in regulating inflammation and oxidative stress during follicular atresia.

### 4.3. Substance Transport and Signal Transduction

Substance transport and signal transduction processes are crucial for cells, as they are essential for cellular metabolic activities, maintaining homeostasis and coordinating cellular responses and communication. We observed KCNG4 (potassium voltage-gated channel modifier subfamily G member 4), which is primarily responsible for the transmembrane transport of potassium ions within cells, and RABGEF1 (Rab guanine nucleotide exchange factor 1), which promotes the activation of small GTPases of the Rab family that regulate the processes of vesicle formation, transport, and fusion [40]. Cell proliferation requires precise substance transport, especially in ovarian cells, which are responsible for the synthesis and secretion of steroid hormones. Therefore, piRNAs may affect the cellular homeostasis and secretory function of granulosa and theca cells by participating in the regulation of the cell membrane potential, the concentration of intracellular potassium ions, and the process of vesicle trafficking. Moreover, GLP2R (glucagon-like peptide receptor), a G protein-coupled receptor, and AKAP17B, a member of the A-kinase anchoring protein (AKAP) family, which are both involved in the regulation of the cAMP-PKA signaling pathway, were also highlighted. Among them, GLP2R has been proven to directly inhibit cellular apoptosis [41], while the primary function of the AKAP family is to enable PKA function by anchoring it to specific subcellular regions [42]. These findings indicate that piRNA functions in the process of signal transduction.

### 4.4. The Maintenance of Cellular Structural Integrity

Among our observations, we noticed several genes related to the cell structure, mainly including EPB41 (erythrocyte membrane protein band 4.1) and PATJ (PALS1-associated tight junction-associated protein). EPB41 is a cytoskeleton-related protein that is involved in cellular response to external signals [43]. In ovarian and cancer cells, it was reported to promote apoptosis through mitogen-activated protein kinase signaling [44]. PATJ, on the other hand, interacts with other tight junction proteins, such as occludin and claudin, to closely connect adjacent cells [45]. It can be easily deduced that normal structures and junctions allow cells to retain their regular adhesion, polarity, and migration and receive appropriate survival signals. However, when disrupted, cells may be more susceptible to the induction of apoptotic signals and undergo apoptosis [46]. Therefore, piRNAs may also maintain cell structure and normal physiological functions.

### 4.5. Thoughts on the Methodology

Regarding the analytical method used in this article, we offer two points of consideration and explanation. Firstly, regarding to the piRNA identification, both piRNApredictor and Piano demonstrated the highest prediction counts for piRNA identification. Neither tool relies on genomic annotations nor existing piRNA databases, making them particularly suitable for studying non-model organisms, like pigs. While piRNApredictor specializes in detecting piRNA clusters within genomes, it shows limited capability in identifying dispersed piRNAs, resulting in significantly fewer predictions compared to those obtained through other methods [21,22,23]. Secondly, among the 12 piRNAs selected from our sequencing results, the PCR verification showed that only half of them exhibited significant differences. One of the reasons for this difference may lie in our choice of a reverse transcription method. We used a common microRNA RT kit with poly(A) primers. However, the 3′ terminal of piRNAs exhibits a structure showing methylation of the 2′-hydroxyl-group of ribonucleotides (2′-O-methylation) [47]. This structure inhibits the activity of the polyA-enzyme, thus leading to a decrease in significant amplification efficiency. Therefore, when performing reverse transcription for piRNA, this problem should be thoroughly considered, and the use of a stem-loop primer or special analytical methods could be a more optimal choice [48].

## 5. Conclusions

In summary, the piRNA profiles of porcine follicles were presented for the first time in this paper. Identifying and characterizing piRNAs that are differentially expressed between healthy and atretic follicles and their target genes revealed pathways and processes in which piRNAs may exert their effects. This study unravels the ncRNA regulatory network of apoptosis and follicular atresia and provides insights into the reproductive regulatory mechanisms in pigs, as well as in ovarian pathological studies.

## Figures and Tables

**Table 1:** HiSeq™ 3000 second-generation sequencing data statistics.

Samples	Total	Clean Reads	Mapping Reads Count	Mapping Rate
HF 1	16,159,470	15,164,669	14,862,688	98.01%
HF 2	15,923,735	14,443,413	14,119,933	97.76%
HF 3	18,060,078	16,382,285	16,105,126	98.31%
AF 1	16,707,009	15,279,119	14,948,455	97.84%
AF 2	19,319,035	17,165,699	16,874,407	98.30%
AF 3	18,171,763	16,803,580	16,473,089	98.03%

## Data Availability

The datasets used during the current study are available from the corresponding author upon reasonable request.

## Supplementary Materials

The following supporting information can be downloaded at: https://www.mdpi.com/article/10.3390/biology14060609/s1, Figure S1. Comparison of piRNA quantity between samples. Note: A: piano sample intersection; B: piRNApredictor sample intersection; C: proTRAC sample intersection. Table S1. List of primers used for PCR detection of small RNAs. Table S2. HiSeq™ 3000 second-generation sequencing data statistics. Table S3. The number of piRNA molecules in each sample predicted by three algorithms. Table S4. piRNA sequence. Table S5. Differentially expressed piRNAs that met the criteria of at least two algorithms. Table S6. Target genes related to differentially expressed piRNAs. Table S7. PCR data for piRNAs. Other related data, including raw sequencing data and additional experimental results, can be obtained from the corresponding author upon reasonable request.
