# Supplementary material for: Porcine Ovarian piRNA Dynamics: A Comparative Study During Follicular Atresia
Source: Biology (Basel). 2025 May 26;14(6):609. doi: 10.3390/biology14060609 (PMC12189065; doi:10.3390/biology14060609)
Supplement: Supplementary file 1 [file biology-14-00609-s001.zip › biology-3581244-supplementary.pdf]

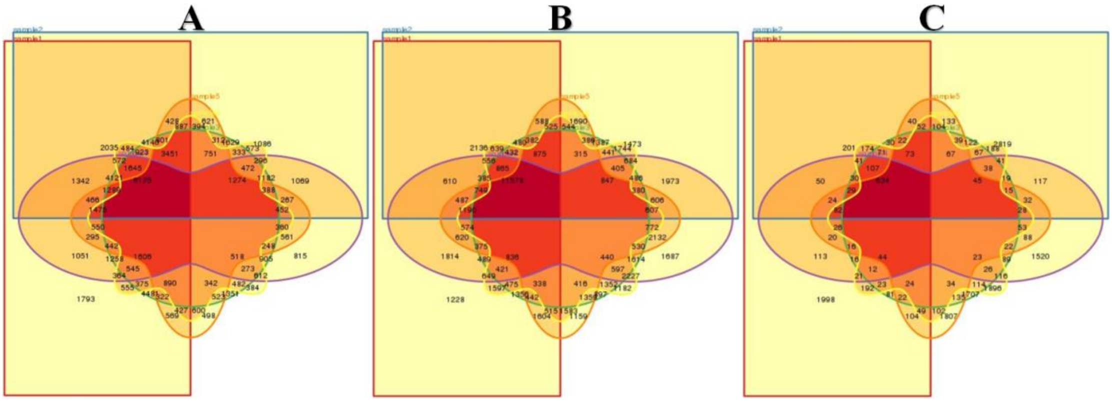

Figure S1. Comparison of piRNA quantity between samples

Note: A: piano sample intersection; B: piRNApredictor sample intersection; C: proTRAC sample intersection

Table S1. List of primers used for PCR detection of small RNAs

piRNA	Primer（5’-3’）
piR-23	TCCATGGTGGTCTAGTGGTTAGGATTC
piR-24	TCCATGGTGGTCTAGTGGTTAGGATTCA
piR-27	TCCATGGTGGTCTAGTGGTTAGGATTT
piR-39	TCCCTGGTGGTCTAATGGTTAGGATTCG
piR-51	TCCCTGGTGGTCTAGTGGTTAGGATC
piR-58	TCCCTGGTGGTCTAGTGGTTAGGATTCA
piR-59	TCCCTGGTGGTCTAGTGGTTAGGATTCC
piR-63	TCCCTGGTGGTCTAGTGGTTAGGATTTG
piR-64	TCCCTGGTGGTCTAGTGGTTAGGGTTC
piR-65	TCCCTGGTGGTCTAGTGGTTGGGATTC
piR-70	TCCCTGGTGGTCTAGTGTTAGGATTCG
piR-76	TCCGTGGTGGTCTAGTGGTTAGGATTCA
U6	GCTTCGGCAGCACATATACT

Table S2. HiSeq ™ 3000 second-generation sequencing data statistics

Samples	Total	5’adapter Contaminats	Removed low quality	Smaller than 17nt	Poly-N/A/C/G	Clean reads
HF 1	16159470	10780	205097	583700	370	15164669
HF 2	15923735	15096	226478	1024160	566	14443413
HF 3	18060078	12998	241863	1176620	209	16382285
AF 1	16707009	18605	239421	1018163	396	15279119
AF 2	19319035	18708	277803	1704129	477	17165699
AF 3	18171763	13612	238191	943244	333	16803580

Table S3. The number of piRNA molecules in each sample predicted by 3 algorithms

algorithm	sample1	sample2	sample3	sample4	sample5	sample6	total
piano	45707	41283	43397	32838	28132	28990	65278
piRNApredictor	35810	36445	32330	38185	35444	36221	67725
proTRAC	4421	5534	3796	3537	3988	4567	16027

Table S4. piRNA sequence

piRNA	Sequence(5'-3')
piR-23	UCCAUGGUGGUCUAGUGGUUAGGAUUC
piR-24	UCCAUGGUGGUCUAGUGGUUAGGAUUCA
piR-27	UCCAUGGUGGUCUAGUGGUUAGGAUUU
piR-39	UCCCUGGUGGUCUAAUGGUUAGGAUUCG
piR-51	UCCCUGGUGGUCUAGUGGUUAGGAUC
piR-58	UCCCUGGUGGUCUAGUGGUUAGGAUUCA
piR-59	UCCCUGGUGGUCUAGUGGUUAGGAUUCC
piR-63	UCCCUGGUGGUCUAGUGGUUAGGAUUUG
piR-64	UCCCUGGUGGUCUAGUGGUUAGGGUUC
piR-65	UCCCUGGUGGUCUAGUGGUUGGGAUUC
piR-70	UCCCUGGUGGUCUAGUGUUAGGAUUCG
piR-76	UCCGUGGUGGUCUAGUGGUUAGGAUUCA

Table S5 Differentially expressed piRNAs that met the criteria of at least two algorithms

(Excel file)

Table S6 Target genes related to differentially expressed piRNAs

(Excel file)

Table S7 PCR data for piRNAs

((Excel files)

Other related data, including raw sequencing data and additional experimental results can be obtained from the corresponding author upon reasonable request.
